# Supplementary figures and images for: Role of miR-30a-3p Regulation of Oncogenic Targets in Pancreatic Ductal Adenocarcinoma Pathogenesis
Source: Int J Mol Sci. 2020 Sep 4;21(18):6459. doi: 10.3390/ijms21186459 (PMC7555373; doi:10.3390/ijms21186459)

ITGA2

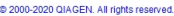

Supplement: Supplementary file 1 [file ijms-21-06459-s001.zip › Supplementary Files/Figure S1.pdf]

ITGA2

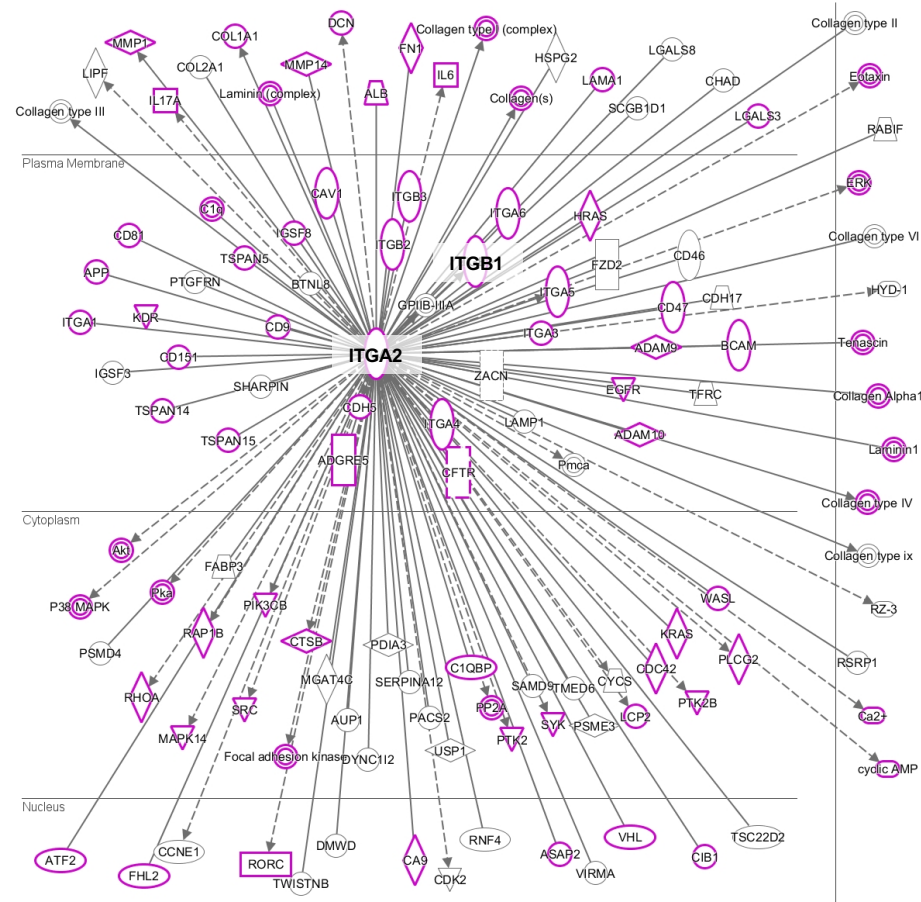

ITGA2

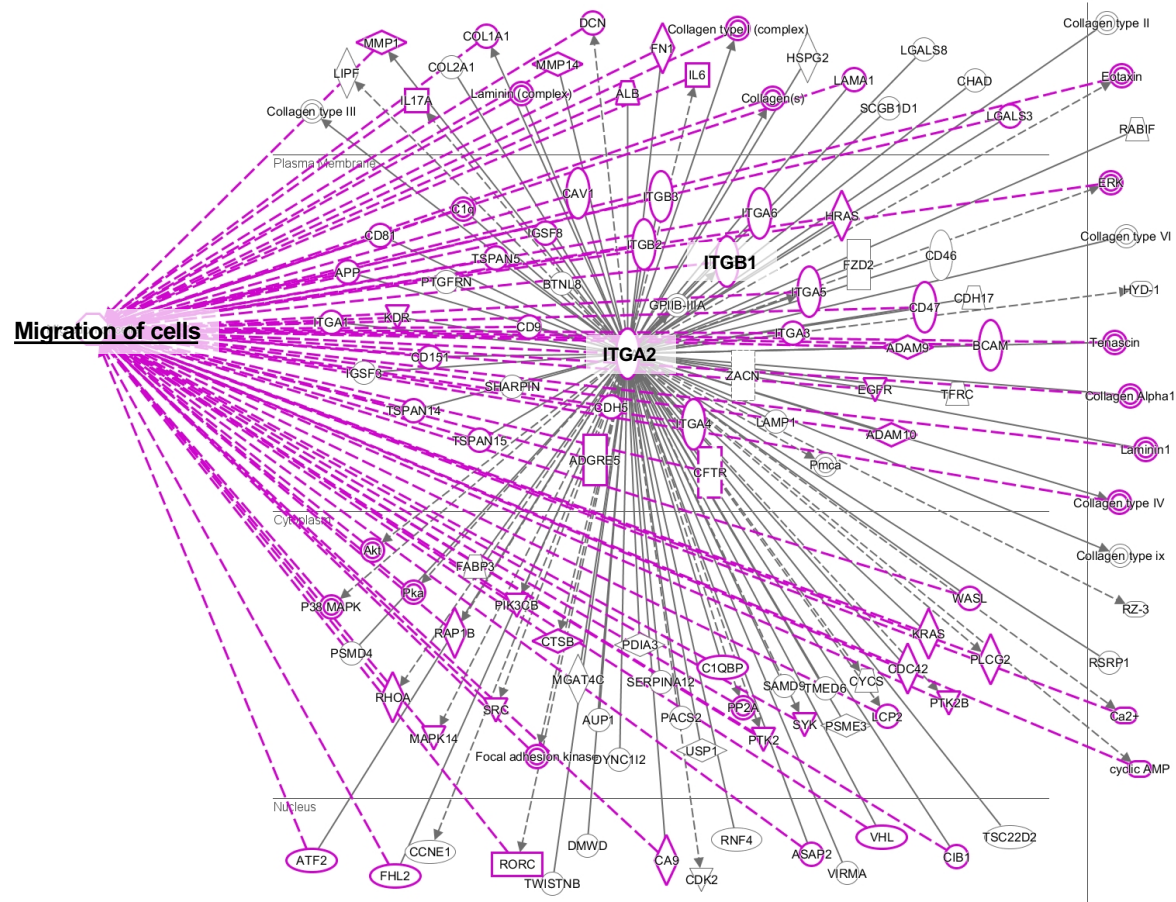

Supplement: Supplementary file 1 [file ijms-21-06459-s001.zip › Supplementary Files/Figure S2.pdf]

ITGA2

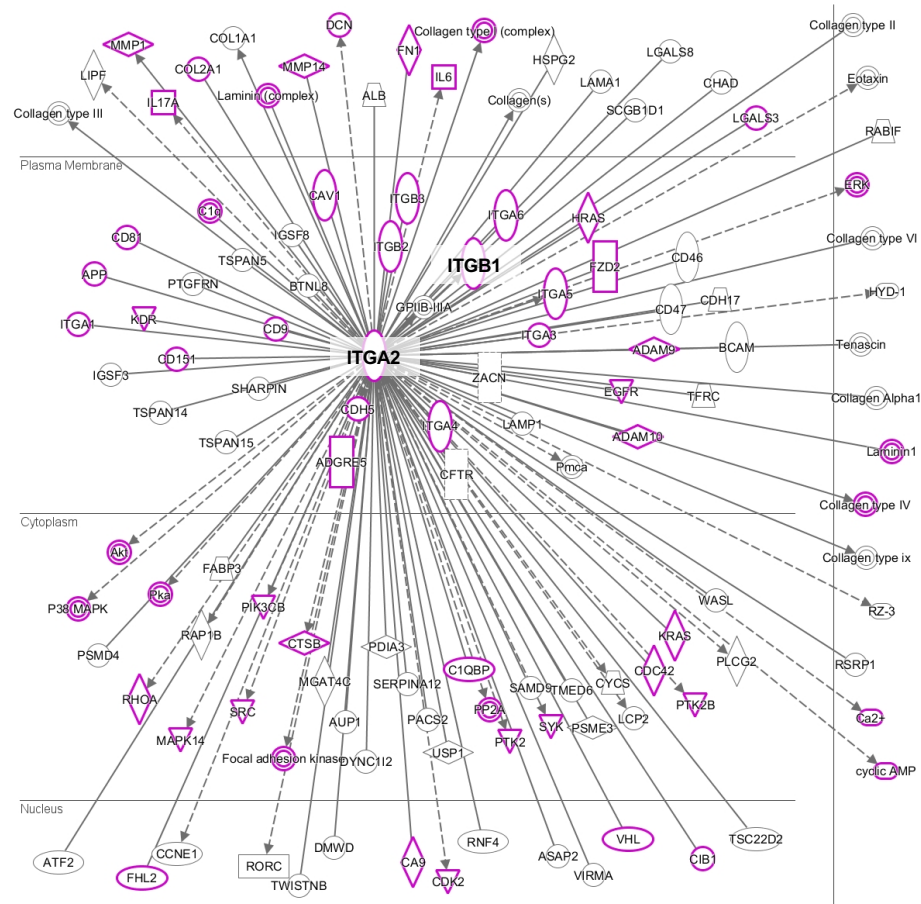

ITGA2

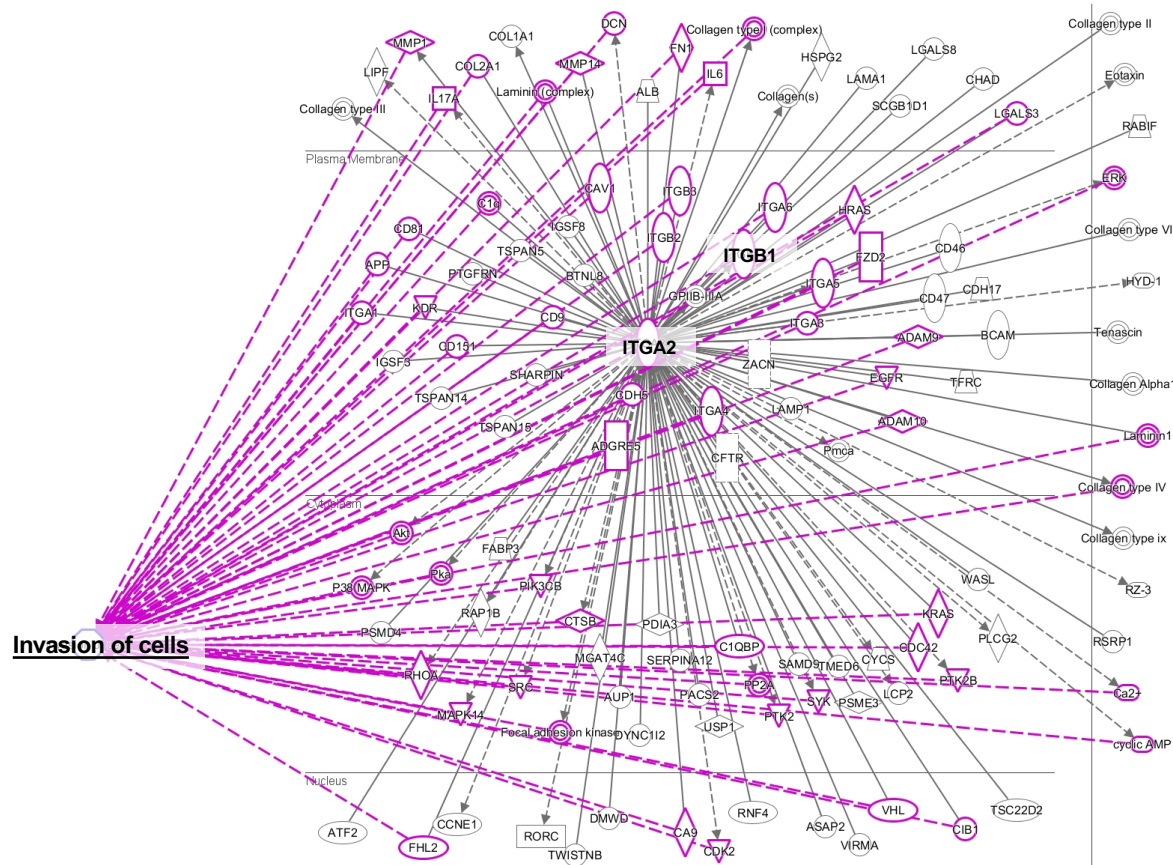

Supplement: Supplementary file 1 [file ijms-21-06459-s001.zip › Supplementary Files/Figure S3.pdf]
